# Concomitant Atrial Fibrillation Radiofrequency Ablation During Total Thoracoscopic Valve Replacement: Safety, Early-Term Efficacy, and Predictors of Early Atrial Arrhythmia Recurrence

**DOI:** 10.1155/cdr/8872143

**Published:** 2025-02-17

**Authors:** Zhiqin Lin, Zheng Xu, Liangwan Chen, Xiaofu Dai

**Affiliations:** ^1^Department of Cardiovascular Surgery, Fujian Provincial Center for Cardiovascular Medicine, Union Hospital, Fujian Medical University, Fuzhou, China; ^2^Key Laboratory of Cardio-Thoracic Surgery, Fujian Medical University, Fujian Province University, Fuzhou, China

**Keywords:** atrial arrhythmia recurrence, atrial fibrillation, minimally invasive cardiac surgery, radiofrequency ablation, total thoracoscopic valve replacement

## Abstract

**Background:** Atrial fibrillation (AF) complicates cardiac surgery, including valve replacements, increasing perioperative risk and impacting long-term outcomes. Concomitant radiofrequency ablation (RFA) during cardiac surgeries shows promise for managing AF. This study investigates the safety, early efficacy, and predictors of atrial arrhythmia recurrence (AAR) following AF RFA during total thoracoscopic valve replacement (TTVR).

**Methods:** This retrospective observational study included 625 patients who underwent TTVR with concomitant AF RFA from January 2017 to May 2023. Demographic data, preoperative characteristics, operative details, and postoperative outcomes were collected. The primary outcome was AAR within 3 months postoperatively.

**Results:** Of the 625 patients, AAR was observed in 21.6% (135 patients), with a median time to recurrence of 45 days. Independent predictors of early AAR included age, AF duration, body mass index (BMI), AF type, left atrial diameter, and ablation extent. Notably, persistent and long-standing persistent AF, a larger left atrial diameter, and ablation of the left atrium alone were associated with higher recurrence risks. The in-hospital mortality rate was 1.6%, with no significant differences in early complications between the recurrence and nonrecurrence groups.

**Conclusions:** Concomitant AF RFA during TTVR is a safe and effective strategy for managing AF in minimally invasive valve surgery. Early predictors of AAR include age, AF duration, BMI, AF type, left atrial diameter, and ablation extent. Future multicenter studies with longer follow-ups are needed to validate these findings and provide robust evidence on long-term outcomes.

## 1. Introduction

Atrial fibrillation (AF), the most common sustained cardiac arrhythmia, significantly complicates the clinical course of patients undergoing cardiac surgery, including valve replacements [1]. Its presence increases not only perioperative risk but also negatively impacts long-term survival and quality of life [2, 3]. Current clinical guidelines recommend concomitant surgical ablation for patients with AF undergoing cardiac surgery [4]. Effective AF management is a critical component of surgical planning and execution. The introduction of concomitant radiofrequency ablation (RFA) during cardiac surgeries, such as valve replacements, has shown promise in managing AF and may help reduce arrhythmia recurrence, thereby improving overall outcomes [5].

Total thoracoscopic valve replacement (TTVR) offers a minimally invasive alternative to traditional sternotomy, potentially reducing recovery times and complications such as infection or bleeding [6]. However, the feasibility and safety of the combination of this technique with RFA for AF ablation have not been extensively studied. While the efficacy of RFA in managing AF during open cardiac procedures is well-documented [7], its adaptation and influencing factors in thoracoscopic procedures require thorough investigation due to the unique challenges and technical nuances involved. Identifying predictors of atrial arrhythmia recurrence (AAR) following concomitant RFA during TTVR is critical for optimizing patient selection, risk stratification, and postoperative care.

This study investigates the safety, early efficacy, and predictors of recurrence of concomitant AF ablation during TTVR. Using a retrospective observational design, we aim to elucidate the integration of these treatment modalities, focusing on early postoperative outcomes and identifying factors influencing the success of AF management in this setting. By addressing a critical gap in the current literature, this research seeks to inform clinical practices by identifying potential predictors of early AAR, thereby improving patient stratification and treatment tailoring.

## 2. Materials and Methods

### 2.1. Study Design and Population

This retrospective observational study included a cohort of 625 consecutive patients who underwent TTVR with concomitant AF RFA at our institution between January 2017 and May 2023. Eligible participants were adults (≥ 18 years) diagnosed with valve disease requiring surgical intervention and persistent or paroxysmal AF. Exclusion criteria included prior valve surgeries, nonthoracoscopic approaches, and lack of patient consent for data use in research. The study received approval from the Institutional Review Board (IRB), and the requirement for informed consent was waived owing to the retrospective nature of the study.

### 2.2. Data Collection and Outcome Measures

Patient data were collected from electronic medical records, including demographics, preoperative characteristics, operative details, and postoperative outcomes. Preoperative data included age, sex, body mass index (BMI), type and duration of AF, left atrial diameter, left ventricular ejection fraction (LVEF), and comorbidities (e.g., hypertension, diabetes, and coronary artery disease). Operative data included the type of valve replaced, concomitant procedures, and procedural times. Follow-up evaluations were conducted at 1, 4, 8, and 12 weeks postoperatively through outpatient visits or telephone interviews.

The primary outcome was AAR, defined as any recurrence of AF, atrial flutter, or atrial tachycardia episodes lasting longer than 30 s, detected by electrocardiogram, Holter monitoring, or implantable loop recorder. Early AAR was defined as AAR occurring within 3 months postoperatively. Secondary outcomes included early postoperative complications, including stroke, pacemaker implantation, and mortality.

### 2.3. Surgical Procedures and Postsurgical Treatment

All surgical procedures were performed by a team of experienced cardiothoracic surgeons in thoracoscopic cardiac surgery. The surgical technique for TTVR with concomitant AF RFA followed previously published protocols established by our group [8]. Patients underwent TTVR with concomitant AF RFA using bipolar RFA forceps and a monopolar radiofrequency pen. Left atrial ablation was performed during a cardiac standstill, while right atrial ablation was conducted after the heart resumed beating. The decision to perform right atrial ablation was based on the preoperative assessment.

After the RFA procedure, valve replacement surgery was performed, with the choice of valve type (bioprosthetic or mechanical) based on the patient's age and shared decision-making. For patients undergoing concomitant aortic valve surgery, the approach made a 1 cm camera incision at the anterior axillary line and a 3-4 cm main working port via anterior minithoracotomy in the third intercostal space. The aortic valve was replaced with either a mechanical or bioprosthetic valve, based on the patient's preference and clinical indications. For isolated mitral and tricuspid valve surgeries, patients underwent thoracoscopic procedures with a 1 cm camera incision at the axillary midline and a 3–4 cm main working port via anterolateral minithoracotomy in the fourth intercostal space. Concomitant tricuspid valve annuloplasty was performed in patients with tricuspid regurgitation of moderate or greater severity.

Postoperative management included tailored medications and anticoagulation therapy based on the type of valve implanted. All patients underwent transthoracic echocardiography before discharge and during follow-up visits. Patients with bioprosthetic valve replacements were prescribed oral anticoagulants (international normalized ratio (INR): 1.7–2.3) for 3 months, while those with mechanical valve replacements were prescribed lifelong anticoagulants (INR: 1.7–2.3). Intravenous amiodarone was administered intraoperatively (150 mg bolus, followed by 1 mg/kg/h for 24 h, then 0.5 mg/kg/h for 48 h, until oral intake was initiated). Subsequently, patients received oral amiodarone (200 mg twice daily for 2 weeks, then 200 mg daily) for 3 months postoperatively. For some patients, rate control was managed with digoxin, beta-blockers, or nondihydropyridine calcium channel blockers, depending on symptoms, hemodynamic status, presence of heart failure, and AF precipitants.

### 2.4. Statistical Analysis

Continuous variables were expressed as mean ± standard deviation or median (interquartile range (IQR)) and compared using Student's *t*-test or Mann–Whitney *U* test, as appropriate. Categorical variables were presented as frequencies and percentages and compared using the chi-square test or Fisher's exact test. Fine and Gray competing risk regression, with death considered a competing risk, was employed to identify independent predictors of early AAR [9]. Covariates were selected based on their clinical relevance and potential impact on early AAR. The subdistribution hazard ratio (HR) and 95% confidence interval (CI) were reported for each covariate. The time-dependent area under the curve (AUC) was calculated to assess the model's predictive accuracy over time. A *p* value of < 0.05 was considered statistically significant. Statistical analyses were conducted using SPSS Version 26.0 (IBM SPSS Inc., Armonk, NY, United States) and R Version 4.4.0 (R Foundation for Statistical Computing, Vienna, Austria).

## 3. Results

### 3.1. Baseline Characteristics

Table 1 summarizes the baseline demographic and clinical characteristics of the study population. A total of 625 patients were included in this study, with a mean age of 56 years (range 48–63). The majority of patients were male (42.6%) and had a history of persistent or long-standing AF (72%). The median AF duration was 2 years (IQR 1–5 years). Other comorbidities included hypertension (19.7%), diabetes (8.6%), and coronary artery disease (7.4%). Echocardiographic parameters revealed a mean left atrial diameter of 45.2 cm (IQR 39.2–49.1 cm) and a mean LVEF of 53.7% ± 9.3%.

During the follow-up period, AAR occurred in 135 patients (21.6%), corresponding to a successful AF management rate of 78.4%. Patients in the recurrence group had a significantly higher BMI (25.8 ± 5.6 vs. 22.7 ± 3.4 kg/m^2^, *p* < 0.001) and a higher prevalence of hypertension (25.9% vs. 18.0%, *p* = 0.039) than those in the nonrecurrence group. The recurrence group also had a higher proportion of long-standing persistent AF (54.8% vs. 35.5%, *p* < 0.001), a longer AF duration (median: 4 vs. 2 years, *p* < 0.001), and a larger left atrial diameter (median: 49.1 vs. 43.9 cm, *p* < 0.001).

### 3.2. Operative Data and Early Outcomes

Operative details, in-hospital outcomes, and postoperative medical treatment are presented in Table 2. The mean cardiopulmonary bypass time and cross-clamp time were 159 (IQR 145–168) and 122 min (IQR 104–128), respectively. The total ablation procedure time was significantly shorter in the recurrence group (31 min, IQR 24–40) than in the nonrecurrence group (39 min, IQR 29–44) (*p* < 0.001). The recurrence group had a significantly higher proportion of patients who underwent left atrial ablation only (55.6% vs. 33.9%, *p* < 0.001), whereas the nonrecurrence group had a higher proportion of patients who underwent biatrial ablation (66.1% vs. 44.4%, *p* < 0.001). The majority of patients underwent mitral valve replacement (85.6%), followed by aortic valve replacement (17.6%). Concomitant procedures, such as tricuspid valve repair or replacement, were performed in a subset of patients.

In-hospital mortality occurred in 10 of 625 patients (1.6%). The causes of death included postoperative low cardiac output syndrome in four patients, severe pulmonary infection leading to multiple organ failure in three patients, cerebrovascular events in two patients, and acute kidney failure in one patient. Among the two patients who experienced fatal cerebrovascular events, one had a documented recurrence of atrial arrhythmia prior to the event, while the other had no recorded recurrence before the cerebrovascular event. There was no significant difference in mortality rates between the recurrence and nonrecurrence groups (1.5% vs. 1.6%, *p* = 0.999). Similarly, there were no significant differences in intensive care unit stay, postoperative hospital stay, or in the incidence of early complications such as respiratory complications, prolonged ventilation, blood loss, or cardiocerebral events.

Postoperatively, a significantly lower proportion of patients in the recurrence group received Class III antiarrhythmic drugs than in the nonrecurrence group (89.6% vs. 96.5%, *p* = 0.001). The pacemaker implantation rate within the first 3 months postoperatively was 0.5% (3/615) among surviving patients, with all cases occurring in the nonrecurrence group. The reasons for pacemaker implantation included sinus node dysfunction in one patient and complete atrioventricular block in two patients.

### 3.3. AAR and Predictors

**Figure 1:**
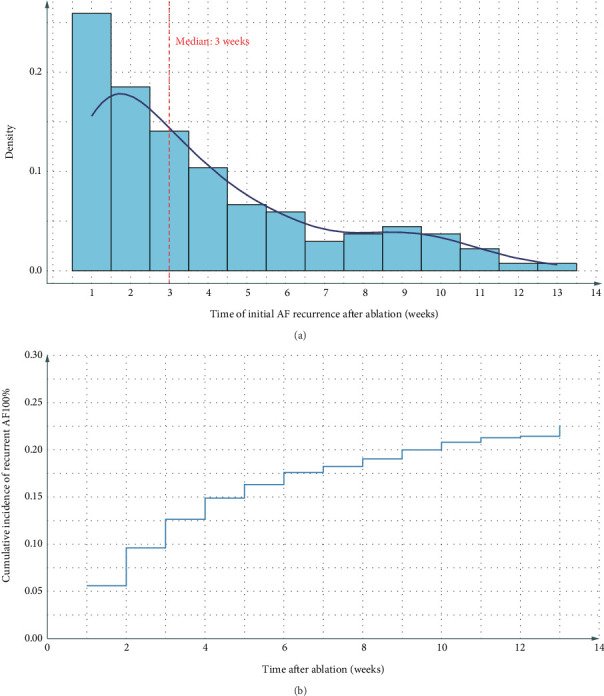
Time distribution of the first postoperative atrial fibrillation occurrence and the cumulative incidence of recurrent atrial arrhythmias during the 3-month follow-up period. (a) The bar graph represents the percentage of patients who experienced their first postoperative atrial fibrillation episode at various time points within the 3-month follow-up period. (b) The line graph illustrates the cumulative incidence of recurrent atrial arrhythmias over the same period.

Figures 1(a) and 1(b) present the time distribution of the first postoperative AAR (Figure 1(a)) and the cumulative incidence of AAR (Figure 1(b)) over the 3-month follow-up period. At the 3-month follow-up, 78.4% (490/625) of patients remained free from early AAR, whereas the cumulative incidence of AAR was 21.6% (135/625). Among patients who experienced recurrence, the median time to recurrence was 45 days (IQR: 30–60 days).  Figure 1 illustrates the cumulative incidence of AAR over the 3-month follow-up period.

**Figure 2:**
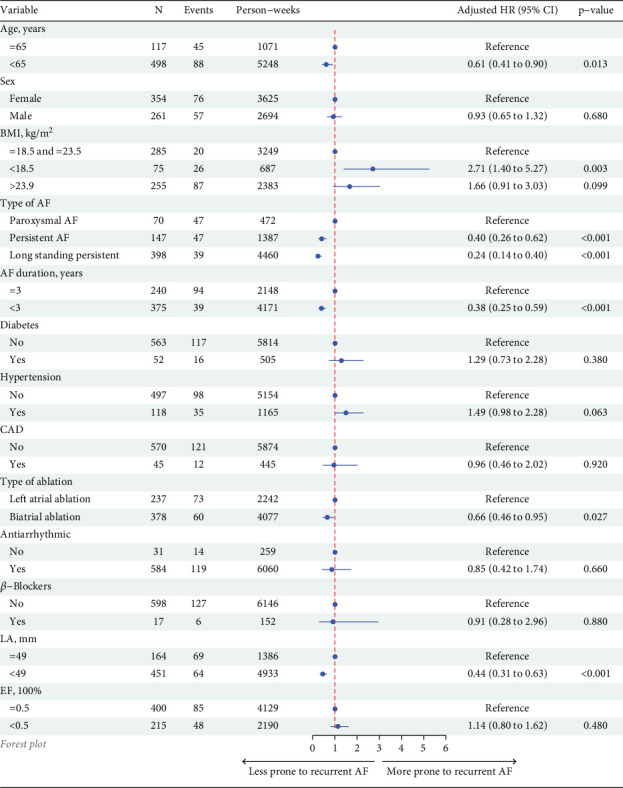
Forest plot of independent predictors of atrial arrhythmia recurrence identified by the Fine–Gray competing risk regression analysis.

The Fine–Gray competing risk regression analysis identified several independent predictors of early AAR (Figure 2). Age was a significant factor, with patients under 65 years exhibiting a reduced risk of early AAR (adjusted HR: 0.68, 95% CI: 0.47–0.99, *p* = 0.043). AF duration was also pivotal, as patients with an AF duration of less than 3 years demonstrated a lower risk of recurrence (adjusted HR: 0.35, 95% CI: 0.23–0.53, *p* < 0.001). BMI displayed a U-shaped relationship with AAR risk; both patients with a BMI less than 18.5 kg/m^2^ (adjusted HR: 2.60, 95% CI: 1.38–4.90, *p* = 0.003) and those with a BMI greater than 23.9 kg/m^2^ (adjusted HR: 2.75, 95% CI: 1.61–4.69, *p* < 0.001) exhibited increased risks of recurrence.

The type of AF emerged as a significant predictor of AAR. Compared to paroxysmal AF, both persistent AF (adjusted HR: 1.80, 95% CI: 1.03–3.16, *p* = 0.040) and long-standing persistent AF (adjusted HR: 1.98, 95% CI: 1.15–3.38, *p* = 0.013) were associated with higher recurrence risks. Additionally, left atrial diameter (HR: 1.06, 95% CI: 1.04–1.09, *p* < 0.001) and left atrial ablation only (HR: 1.98, 95% CI: 1.41–2.78, *p* < 0.001) were linked to increased AAR risk. Validation of the model yielded an AUC of 0.858 (95% CI: 0.785–0.931) at 3 months.

## 4. Discussion

This retrospective observational study provides valuable insights into the safety, early-term efficacy, and predictors of recurrence of concomitant AF RFA during TTVR. Our findings indicate that this combined approach is both safe and effective for managing AF in patients undergoing minimally invasive valve surgery, with a 78.4% success rate in maintaining sinus rhythm at the 3-month follow-up. Additionally, we identified several factors associated with the risk of early AAR, including age, AF duration, BMI, AF type, left atrial diameter, and the extent of ablation.

The safety of concomitant AF RFA during TTVR is supported by our study's findings, which are consistent with previous reports on open cardiac surgeries [10]. The in-hospital mortality rate of 1.6% is comparable to those reported in studies examining concomitant AF ablation during conventional valve surgeries, which range from 1.2% to 3.2% [10, 11]. Furthermore, the incidence of early complications, including respiratory complications, prolonged ventilation, blood loss, and cardiocerebral events, did not significantly differ between the recurrence and nonrecurrence groups, suggesting that the addition of RFA to TTVR does not increase the risk of these adverse events. The minimally invasive thoracoscopic approach may contribute to this favorable safety profile, as it has been shown to reduce surgical trauma and postoperative complications compared to traditional sternotomy [12].

The early-term efficacy of concomitant AF RFA during TTVR in our study aligns with the success rates reported in the literature for open cardiac surgeries. A meta-analysis by Cheng et al. found that the number of patients in sinus rhythm was significantly improved at discharge in the surgical AF ablation group (68.6%) [13]. Our study's success rate of 78.4% at 3 months is promising, particularly given the minimally invasive nature of TTVR and the shorter follow-up period. However, longer-term follow-up is necessary to assess the durability of these results and compare them with those of open cardiac surgeries.

In a retrospective observational study involving 625 cases, we identified key predictors of early AAR following concomitant AF RFA during TTVR. Patients under 65 years exhibited a reduced risk of AAR, consistent with previous studies that have identified advanced age as a risk factor for AF recurrence after ablation. MacGregor et al. specifically examined the impact of age on AF recurrence following surgical ablation and concluded that age was a significant predictor of atrial tachyarrhythmia recurrence. They observed that the efficacy of the Cox-Maze IV procedure, a widely used surgical ablation technique, was lower in elderly patients [14]. The association between age and AF recurrence may be attributed to age-related structural and electrical remodeling of the atria, which promotes arrhythmogenesis. These findings, along with our results, underscore the importance of considering age as a factor in patient selection and outcome prediction for concomitant AF ablation during cardiac surgery. A duration of AF of less than 3 years was associated with a lower risk of recurrence, aligning with the concept that longer AF duration leads to more extensive atrial remodeling and treatment resistance. This finding is corroborated by Charitos et al., who found preoperative AF duration to be independently associated with higher postoperative AF burden and recurrence [15]. These results highlight the importance of AF duration as a predictor of ablation success and emphasize the need for early intervention in AF management. Timely surgical ablation may lead to better outcomes by addressing the arrhythmia before extensive atrial remodeling develops. A novel U-shaped correlation between BMI and AAR risk was observed, emphasizing the necessity for meticulous patient selection and tailored treatment strategies. Specifically, patients at both extremes of BMI (< 18.5 and > 23.9 kg/m^2^) exhibited heightened recurrence risks, potentially due to associated metabolic and structural alterations. This finding is supported by Liu et al., who reported that elevated BMI increased the risk of AF recurrence by 31% [16], and by Deng et al., who found a U-shaped relationship between BMI and AF recurrence postablation, noting that both underweight and overweight individuals are at increased risk [17]. Persistent and long-standing persistent AF was associated with higher recurrence risks compared to paroxysmal AF. This finding aligns with the progressive nature of AF and the increasing difficulty in maintaining sinus rhythm as the disease progresses. This observation is consistent with the conclusions of Mesquita et al., who identified nonparoxysmal AF as an independent predictor in their risk score model for predicting AF recurrence after the first catheter ablation procedure [18]. Left atrial diameter and ablation strategy were also identified as independent predictors of AAR. Patients with larger left atrial diameters had a higher risk of recurrence, consistent with previous studies showing left atrial size as a predictor of AF recurrence after ablation. Sunderland, Maruthappu, and Nagendran found that patients with a mean preoperative left atrial diameter greater than 60 mm should be approached with caution when considering maze procedures [19], highlighting the importance of left atrial size in AF treatment outcomes. The finding that left atrial ablation alone was associated with a higher risk of recurrence compared to biatrial ablation suggests that a more comprehensive ablation strategy may be beneficial for certain patient populations. This is in line with the findings of Cappabianca et al., who concluded that concomitant biatrial surgical ablation appears superior to left atrial ablation in terms of efficacy, even though their study focused on open surgical ablation rather than minimally invasive thoracoscopic procedures [20].

In terms of the impact of different valve replacement surgeries on postoperative AAR, there is currently no relevant research specifically examining this issue. Valve surgeries involve a wide range of procedures, including the number of valves involved, different valve types (such as mitral, aortic, or tricuspid valve replacement), and whether the surgery involves valve repair or replacement. These factors may influence atrial remodeling and electrophysiological properties, which could potentially affect the risk of arrhythmia recurrence. Due to the complexity and variety of these procedures, this remains an area largely unexplored in the literature. Future studies should investigate the relationship between valve surgery types and AAR recurrence, focusing on how different surgical approaches and ablation strategies may impact the long-term outcome of AF management.

The study's findings provide valuable insights into the management of AF in patients undergoing TTVR and advocate for a comprehensive, interdisciplinary approach. The identified predictors of early AAR can inform patient selection, risk stratification, and personalized postoperative care strategies to optimize outcomes [21]. These results contribute to the growing evidence supporting the integration of RFA in minimally invasive cardiac surgeries, underscoring the importance of considering multiple factors for optimal patient management and the potential benefits of concomitant AF treatment during TTVR.

The main limitations of our study include its retrospective design, which may introduce selection and information biases, as well as the relatively short follow-up period of 3 months. Additionally, the single-center nature of the study may limit the generalizability of the findings. Future prospective, multicenter studies with longer follow-up periods are needed to validate our results and provide more robust evidence on the long-term outcomes of concomitant AF RFA during TTVR.

## 5. Conclusion

In conclusion, our study demonstrates that concomitant AF RFA during TTVR is a safe and effective strategy for managing AF in patients undergoing minimally invasive valve surgery. We identified several predictors of early AAR, including age, AF duration, BMI, AF type, left atrial diameter, and the extent of ablation. These findings provide valuable insights for patient selection, risk stratification, and postoperative management in the context of TTVR with concomitant AF RFA. Further research is warranted to evaluate long-term outcomes and compare the efficacy of various ablation strategies in this setting.

## Figures and Tables

**Table 1:** Comparison of patients' baseline demographic and clinical characteristics.

**Variables** ^**a**^	**Total sample (** **n** = 625**)**	**Patient groups**		
		**Nonrecurrence (** **n** = 490**)**	**Recurrence (** **n** = 135**)**	**p** ** value**
Age (years)	56 (48~63)	56 (49~62)	54 (37.5~68)	0.265
Male, *n* (%)	266 (42.6%)	209 (42.7%)	57 (42.2%)	0.928
BMI (kg/m^2^)	23.4 ± 4.2	22.7 ± 3.4	25.8 ± 5.6	< 0.001
Smoking history, *n* (%)	104 (16.6%)	25 (18.5%)	79 (16.1%)	0.508
Comorbidities				
Diabetes, *n* (%)	54 (8.6%)	38 (7.8%)	16 (11.9%)	0.134
Hypertension, *n* (%)	123 (19.7%)	88 (18.0%)	35 (25.9%)	0.039
COPD, *n* (%)	21 (3.4%)	15 (3.1%)	6 (4.4%)	0.603
CAD, *n* (%)	46 (7.4%)	34 (6.9%)	12 (8.9%)	0.442
Stroke history, *n* (%)	37 (5.9%)	32 (6.5%)	5 (3.7%)	0.218
Liver dysfunction, *n* (%)	64 (10.2%)	51 (10.4%)	13 (9.6%)	0.792
Dialysis, *n* (%)	14 (3.93%)	4 (2.37%)	10 (5.35%)	0.241
Peripheral vascular disease, *n* (%)	17 (2.7%)	12 (2.5%)	5 (3.7%)	0.621
Cancer history, *n* (%)	14 (2.2%)	12 (2.4%)	2 (1.5%)	0.731
Type of AF, *n* (%)				
Paroxysmal AF	175 (28.0%)	157 (32.0%)	18 (13.3%)	< 0.001
Persistent AF	202 (32.3%)	159 (32.5%)	43 (31.9%)	
Long-standing persistent, *n* (%)	248 (39.7%)	174 (35.5%)	74 (54.8%)	
AF duration, years	2 (1~5)	2 (1~4)	4 (2~6)	< 0.001
NYHA III/IV, *n* (%)	16 (4.49%)	6 (3.55%)	10 (5.35%)	0.575
Echocardiographic parameters				
Severe pulmonary hypertension, *n* (%)	145 (23.2%)	113 (23.7%)	37 (5.9%)	0.876
Left atrium diameter (cm)	45.2 (39.2~49.1)	43.9 (38.725~48.2)	49.1 (45.15~54.7)	< 0.001
LVEF (%)	53.7 ± 9.3	53.8 ± 9.2	53.1 ± 9.7	0.416

Abbreviations: AF, atrial fibrillation; CAD, coronary artery disease; COPD, chronic obstructive pulmonary disease; LAT, left anterior thoracotomy; LVEF, left ventricular ejection fraction; NYHA, New York Heart Association.

^a^Continuous variables are presented as mean ± standard deviation. Non-normally distributed variables are presented as the median (interquartile range), and categorical data are presented as numbers (percent).

**Table 2:** Operative details, in-hospital outcomes, and postoperative medical treatment of the population.

**Variables** ^**a**^	**Total sample (** **n** = 625**)**	**Patient groups**		
		**Non-recurrence (** **n** = 490**)**	**Recurrence (** **n** = 135**)**	**p** ** value**
Cardiopulmonary bypass (minutes)	159 (145~168)	159 (146~168)	158 (139~166)	0.122
Cross-clamp (minutes)	122 (104~128)	122 (105~128)	123 (101~128)	0.486
Total ablation procedure time (minutes)	37 (28~43)	39 (29~44)	31 (24~40)	< 0.001
Extent of ablation				
Left atrial ablation, *n* (%)	241 (38.6%)	166 (33.9%)	75 (55.6%)	< 0.001
Biatrial ablation, *n* (%)	384 (61.4%)	324 (66.1%)	60 (44.4%)	
Valve procedure, *n* (%)				
AVR	110 (17.6%)	89 (18.2%)	21 (15.6%)	0.481
MVR	535 (85.6%)	420 (85.7%)	115 (85.2%)	0.877
MVP				
DVR^b^	93 (14.9%)	76 (15.5%)	17 (12.6%)	0.399
TVR	17 (2.7%)	15 (3.1%)	2 (1.5%)	0.484
TVP	121 (19.4%)	88 (18.0%)	33 (75.6%)	0.091
Concomitant LAT	50 (8.0%)	38 (7.8%)	12 (8.9%)	0.667
Hospital mortality, *n* (%)	10 (1.6%)	8 (1.6%)	2 (1.5%)	> 0.999
Intensive care unit stay (days)	2 (2~3)	2 (2~3)	3 (2~4)	0.604
Postoperative hospital stay (days)	9 (8~11)	9 (8~11)	9 (8~11)	0.372
Early complications				
Respiratory complication, *n* (%)	209 (33.4%)	166 (33.9%)	43 (31.9%)	0.446
Prolonged ventilation, *n* (%)	29 (4.6%)	23 (4.7%)	6 (4.4%)	0.903
Blood loss (first 24 h) (mL)	260 (170~430)	230 (175~395)	270 (170~437.5)	0.153
Cardiocerebral events, *n* (%)	5 (0.8%)	4 (0.8%)	1 (0.7%)	> 0.999
IABP, *n* (%)	2 (0.3%)	2 (0.4%)	0 (0%)	NA
ECMO, *n* (%)	2 (0.3%)	2 (0.4%)	0 (0%)	NA
Postoperative medical treatment^b^				
VKA	615 (100%)	482 (100%)	133 (100%)	NA
Antiarrhythmic drug Class III	594 (95.0%)	473 (96.5%)	121 (89.6%)	0.001
ß-Blockers	17 (2.8%)	11 (2.3%)	6 (4.5%)	0.276
Diuretics	520 (84.6%)	409 (84.9%)	111 (83.5%)	0.693
Statin	81 (13.2%)	59 (12.2%)	22 (16.5%)	0.194
Pacemaker implantation^c^, *n* (%)	0.5% (3/615)	0.6% (3/490)	0% (0/135)	NA

Abbreviations: AVR, aortic valve replacement; DVR, double valve replacement; ECMO, extracorporeal membrane oxygenation; IABP, intra-aortic balloon pump; LAT, left atrial thrombectomy; MVP, mitral valvuloplasty; MVR, mitral valve replacement; NA, not applicable; TVP, tricuspid valvuloplasty; TVR, tricuspid valve replacement; VKA, vitamin K antagonist.

^a^Continuous variables are presented as mean ± standard deviation. Non-normally distributed variables are presented as the median (interquartile range), and categorical data are presented as numbers (percent).

^b^Refers to mitral valve replacement and aortic valve replacement.

^c^Among 615 surviving patients within the first 3 months postoperatively.

## Data Availability

The data that support the findings of this study are available on request from the corresponding authors. The data are not publicly available due to privacy or ethical restrictions.

## References

[1] Banach M., Mariscalco G., Ugurlucan M., Mikhailidis D. P., Barylski M., Rysz J. (2008). The significance of preoperative atrial fibrillation in patients undergoing cardiac surgery: preoperative atrial fibrillation—still underestimated opponent. *Europace*.

[2] Karamchandani K., Khanna A. K., Bose S., Fernando R. J., Walkey A. J. (2020). Atrial fibrillation: current evidence and management strategies during the perioperative period. *Anesthesia and Analgesia*.

[3] Aliot E., Botto G. L., Crijns H. J., Kirchhof P. (2014). Quality of life in patients with atrial fibrillation: how to assess it and how to improve it. *Europace*.

[4] Calkins H., Hindricks G., Cappato R. (2018). 2017 HRS/EHRA/ECAS/APHRS/SOLAECE expert consensus statement on catheter and surgical ablation of atrial fibrillation. *Europace*.

[5] Dominici C., Chello M. (2022). Concomitant surgical ablation for treatment of atrial fibrillation in patients undergoing cardiac surgery. *Reviews in Cardiovascular Medicine*.

[6] Tabata M., Fukui T., Takanashi S. (2013). Do minimally invasive approaches improve outcomes of heart valve surgery?. *Circulation Journal*.

[7] Cheng Y.-T., Huang Y.-T., Tu H.-T. (2023). Long-term outcomes of concomitant surgical ablation for atrial fibrillation. *The Annals of Thoracic Surgery*.

[8] Xu Z., Dai X., Lin F., Chen L., Lin Z. (2023). Two-incision totally thoracoscopic mitral valve repair combined with radiofrequency atrial fibrillation ablation in rheumatic mitral valve disease: early results of a case series of 43 consecutive patients. *International Journal of Cardiology*.

[9] Austin P. C., Fine J. P. (2017). Practical recommendations for reporting Fine-Gray model analyses for competing risk data. *Statistics in Medicine*.

[10] Churyla A., Andrei A.-C., Kruse J. (2021). Safety of atrial fibrillation ablation with isolated surgical aortic valve replacement. *The Annals of Thoracic Surgery*.

[11] Kim W. K., Kim H. J., Kim J. B. (2019). Concomitant ablation of atrial fibrillation in rheumatic mitral valve surgery. *The Journal of Thoracic and Cardiovascular Surgery*.

[12] Liu J., Chen B., Zhang Y. Y. (2019). Mitral valve replacement via minimally invasive totally thoracoscopic surgery versus traditional median sternotomy: a propensity score matched comparative study. *Annals of Translational Medicine*.

[13] Cheng D. C. H., Ad N., Martin J. (2010). Surgical ablation for atrial fibrillation in cardiac surgery a meta-analysis and systematic review. *Innovations*.

[14] MacGregor R. M., Khiabani A. J., Bakir N. H. (2021). Impact of age on atrial fibrillation recurrence following surgical ablation. *The Journal of Thoracic and Cardiovascular Surgery*.

[15] Charitos E. I., Ziegler P. D., Stierle U., Graf B., Sievers H.-H., Hanke T. (2015). Long-term outcomes after surgical ablation for atrial fibrillation in patients with continuous heart rhythm monitoring devices. *Interactive Cardiovascular and Thoracic Surgery*.

[16] Guijian L., Jinchuan Y., Rongzeng D., Jun Q., Jun W., Wenqing Z. (2013). Impact of body mass index on atrial fibrillation recurrence: a meta-analysis of observational studies. *Pacing and Clinical Electrophysiology*.

[17] Deng H., Shantsila A., Guo P. (2018). A U-shaped relationship of body mass index on atrial fibrillation recurrence post ablation: a report from the Guangzhou atrial fibrillation ablation registry. *eBioMedicine*.

[18] Mesquita J., Ferreira A. M., Cavaco D. (2018). Development and validation of a risk score for predicting atrial fibrillation recurrence after a first catheter ablation procedure – ATLAS score. *EP Europace*.

[19] Sunderland N., Maruthappu M., Nagendran M. (2011). What size of left atrium significantly impairs the success of maze surgery for atrial fibrillation?. *Interactive Cardiovascular and Thoracic Surgery*.

[20] Cappabianca G., Ferrarese S., Tutino C. (2019). Safety and efficacy of biatrial vs left atrial surgical ablation during concomitant cardiac surgery: a meta-analysis of clinical studies with a focus on the causes of pacemaker implantation. *Journal of Cardiovascular Electrophysiology*.

[21] Kisheva A., Yotov Y. (2021). Risk factors for recurrence of atrial fibrillation. *Anatolian Journal of Cardiology*.

